# A Radiosensitizing Inhibitor of HIF-1 alters the Optical Redox State of Human Lung Cancer Cells *In Vitro*

**DOI:** 10.1038/s41598-018-27262-y

**Published:** 2018-06-11

**Authors:** David E. Lee, Kinan Alhallak, Samir V. Jenkins, Isaac Vargas, Nicholas P. Greene, Kyle P. Quinn, Robert J. Griffin, Ruud P. M. Dings, Narasimhan Rajaram

**Affiliations:** 1 Department of Biomedical Engineering, University of Arkansas, Fayetteville, AR 72701 USA; 2 Department of Radiation Oncology, University of Arkansas for Medical Sciences, Little Rock, AR 72205 USA; 3 Department of Health, Human Performance, and Recreation, University of Arkansas, Fayetteville, AR 72701 USA

## Abstract

Treatment failure caused by a radiation-resistant cell phenotype remains an impediment to the success of radiation therapy in cancer. We recently showed that a radiation-resistant isogenic line of human A549 lung cancer cells had significantly elevated expression of hypoxia-inducible factor (HIF-1α), and increased glucose catabolism compared with the parental, radiation-sensitive cell line. The objective of this study was to investigate the longitudinal metabolic changes in radiation-resistant and sensitive A549 lung cancer cells after treatment with a combination of radiation therapy and YC-1, a potent HIF-1 inhibitor. Using label-free two-photon excited fluorescence microscopy, we determined changes in the optical redox ratio of FAD/(NADH and FAD) over a period of 24 hours following treatment with YC-1, radiation, and both radiation and YC-1. To complement the optical redox ratio, we also evaluated changes in mitochondrial organization, glucose uptake, reactive oxygen species (ROS), and reduced glutathione. We observed significant differences in the optical redox ratio of radiation-resistant and sensitive A549 cells in response to radiation or YC-1 treatment alone; however, combined treatment eliminated these differences. Our results demonstrate that the optical redox ratio can elucidate radiosensitization of previously radiation-resistant A549 cancer cells, and provide a method for evaluating treatment response in patient-derived tumor biopsies.

## Introduction

Radiation therapy is a critical first line of care that is used to treat the majority of cancer patients^1^. Treatment failures caused by a radiation-resistant cell phenotype remain an impediment to the success of cancer treatment. Hypoxic tumors tend to respond poorly to radiation because DNA damage relies on the presence of oxygen^2^. Tumor reoxygenation following radiation can lead to stabilization of hypoxia-inducible factor (HIF-1)^3,4^, thereby increasing glycolytic metabolism^5^ and further promoting cell survival after radiation^6,7^. As a regulator of oxygen homeostasis, HIF-1α plays a key role in downregulating mitochondrial oxygen consumption and enhancing transcription of important glycolytic genes such as pyruvate dehydrogenase kinase (PDK-1)^8,9^. For this reason, pharmaceutical approaches targeting HIF-1α have shown promise in sensitizing radiation-resistant cancer cells to radiotherapy^10^. Other factors in the tumor microenvironment, such as poor oxygen perfusion can also contribute to elevated levels of HIF-1α, thereby compounding the cellular response to radiation^11^. However, recent evidence suggests that radiation-resistant cancer cells have intrinsically elevated HIF-1α expression and a greater glycolytic phenotype independent of radiation therapy or microenvironmental factors^12^.

Nicotinamide adenine dinucleotide (NADH) and flavin adenine dinucleotide (FAD) are fluorescent metabolic cofactors that play important roles in the major metabolic pathways of the cytoplasm and the mitochondria. Specifically, the optical reduction-oxidation (redox) ratio (ORR) of FAD/(FAD + NADH) provides a validated method to quantify the redox state of a cell^13^. During oxidative phosphorylation, oxidation of NADH to non-fluorescent NAD^+^ and FADH_2_ to fluorescent FAD leads to an increase in the ORR. On the other hand, a decrease in the ORR due to a buildup of NADH that is not converted to NAD^+^ can be produced by conditions that decrease the rate of oxidative phosphorylation, such as hypoxia or conditions that increase the rate of glucose catabolism, such as macromolecular synthesis^14^. Several recent studies have utilized the ORR to determine the relationship between cellular metabolism and metastatic potential^15–17^, and identify metabolic response to radiation^12^ or chemotherapy^18–20^. These studies demonstrate that this non-destructive, label-free imaging approach is a valuable technique for characterizing metabolic reprogramming and has potential clinical application to identify treatment efficacy in tumor-derived organoids. However, we lack an understanding of the time-dependent changes in ORR in response to targeted therapies, either alone or in combination with radiation, and how these changes might manifest in radiation-resistant versus sensitive cells.

The purpose of this investigation was to identify the acute redox changes in radiation-resistant lung cancer cells treated with a radiosensitizing HIF-1α inhibitor. A further goal was to determine if this type of chemotherapy, when combined with radiation exposure, could reverse the optical redox characteristics associated with radiation resistance. Using an isogenic clone of radiation-resistant human A549 cancer cells, we determined the changes in ORR in response to the radiosensitizing HIF-1 inhibitor, YC-1, either alone or in combination with radiation. We also measured other metabolic endpoints, such as glucose uptake, ROS levels, and reduced glutathione to determine their contribution to the redox state. In addition, we utilized a Fourier-based fractal analysis of endogenous fluorescence images of NADH to elucidate changes in mitochondrial organization. Our results demonstrate an increase in the ORR of A549RR cells, indicating a reduction in glucose catabolism, when treated with YC-1. We further show that a combination of YC-1 with radiotherapy can identify changes in redox state of radiation- sensitive and resistant cancer cells. Understanding how changes in the cellular redox state in the post-radiation environment are confounded by drug treatment provides a basis for label-free optical imaging to determine how patients might respond to chemoradiation therapy even before they commence treatment.

## Results and Discussion

### Inhibition of HIF-1α increases the ORR, decreases glucose uptake, and increases ROS production

**Figure 1:**
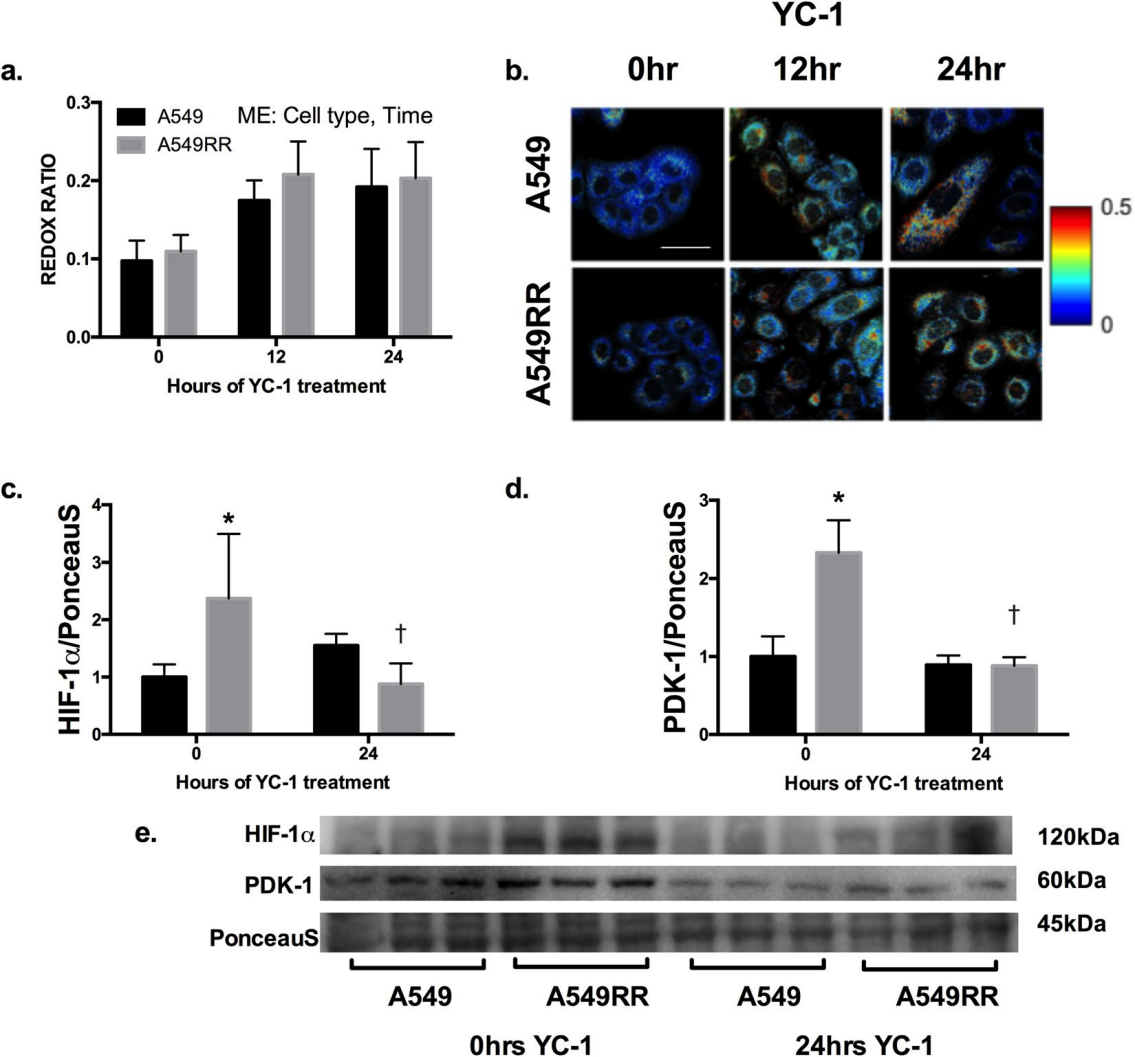
Optical redox ratio (ORR) demonstrates reduced glycolytic phenotype following 24 hours of YC-1 treatment. ORR of A549 and A549RR cells before, 12 and 24 hours following 50 μM YC-1 treatment to inhibit HIF-1 (**a,b**). Immunoblotting for HIF-1α (**c**) and PDK-1 (**d**) before and 24 hours following YC-1 treatment to verify in of HIF-1α. Representative immunoblots were horizontally cropped at the molecular weight indicated (**e**). Uncropped exposures can be seen in Supplemental Fig. 1. Experiments were performed in triplicate across 3 independent experiments. *p < 0.05 compared to A549 0 hr control, ^†^p < 0.05 compared to A549RR 0hrs. Scale bar in images represents 50 μm. ME – Main effect of cell type and time; however, no significant interactions were found between the two main effects.

Depending on the cell type being studied and the specific context of investigation, the optical redox ratio is sensitive to the metabolic pathways co-opted by cells that can produce a relative change in glucose catabolism relative to oxidative phosphorylation^13–15,19–22^. Given our recent results identifying increased HIF-1α in radiation-resistant cells prior to radiation, we used YC-1, a radiosensitizing inhibitor of HIF-1 to determine its effect on the ORR. Both cell type (p = 0.02) and time after YC-1 treatment (p < 0.0001) had statistically significant effects on the optical redox ratio. Following 24 hours of incubation with 50 μM YC-1, the ORR was ~75–100% (p < 0.0001) greater in both A549 and A549RR cells (Fig. 1a,b), indicating greater reliance on oxidative metabolism. There were no significant differences between the cell types at any of the time points. YC-1 successfully downregulated the A549RR HIF-1α content and downstream target PDK-1 to that of the A549 (p > 0.05 at 24hrs) despite starting ~2 fold higher (p < 0.05 at 0hrs, Fig. 1c–e). The inhibition of HIF-1 and subsequent downregulation of PDK-1 are consistent with previous work showing that HIF-1 suppresses mitochondrial oxidative metabolism by inducing PDK-1, which suppresses pyruvate flux through the tricarboxylic acid (TCA) cycle^8,9^.

**Figure 2:**
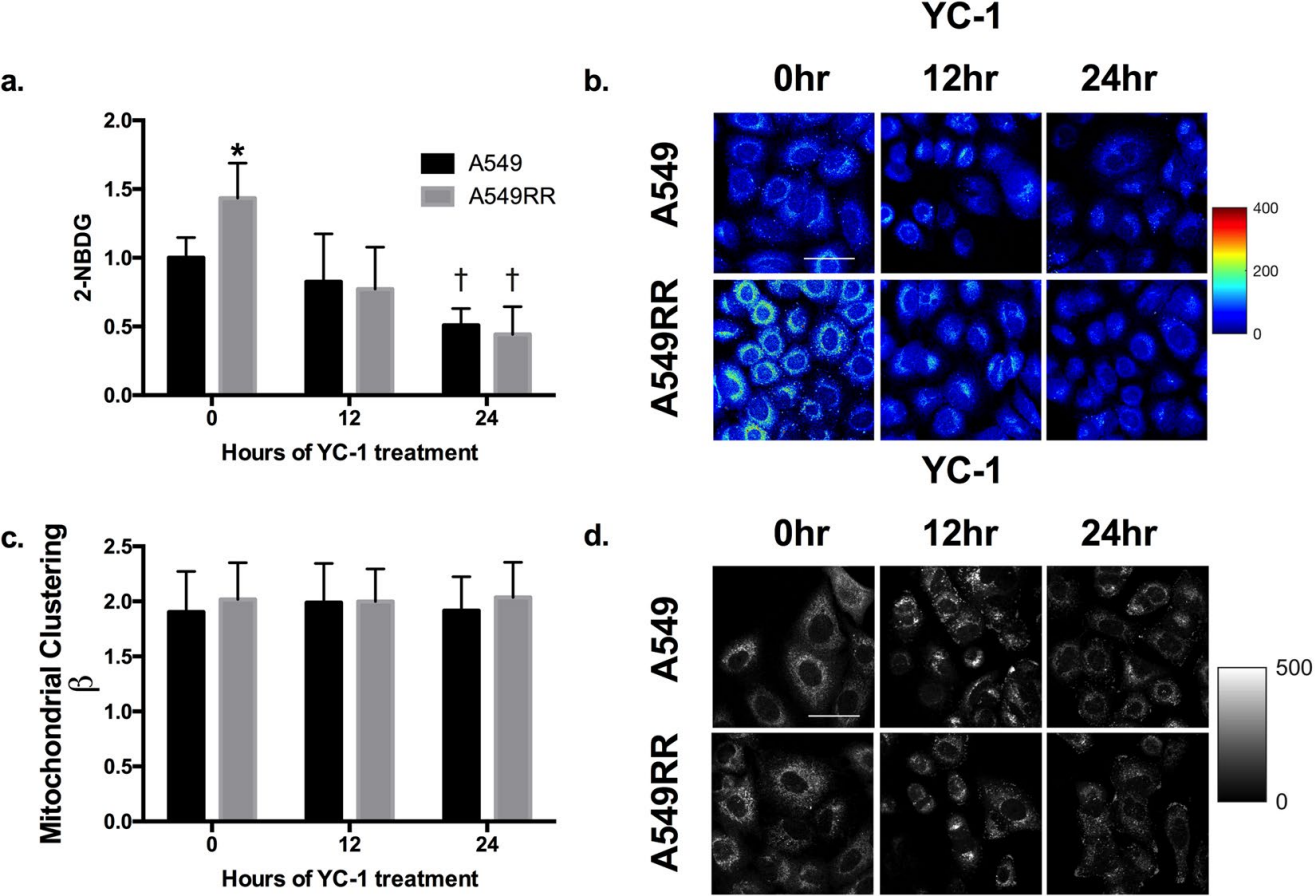
Glucose uptake and mitochondrial clustering following inhibition of HIF-1α. Glucose uptake assessed by 2-NBDG fluorescence in A549 and A549RR cells before, 12 and 24 hours following 50 μM YC-1 treatment (**a,b**). Mitochondrial organization was evaluated through Fourier-based image analysis of the NADH images (**c,d**). *p < 0.05 compared to A549 0 hr control, ^†^p < 0.05 compared to same cell type at 0 hours. Scale bar in images represents 50 μm.

To better understand the metabolic characteristics associated with the YC-1-induced increase in ORR, glucose uptake was measured using the glucose analog 2-NBDG as described previously^23^. A549RR cells had significantly greater 2-NBDG uptake compared with the A549 cells (p < 0.001) prior to YC-1 treatment, thus verifying the effects of HIF-1α elevation on glucose metabolism (Fig. 2a,b). 2-NBDG was downregulated in both cell types in a time-dependent manner (p < 0.001). Specifically, 2-NBDG uptake was decreased by ~50% in the A549 cells and ~70% in the A549RR cells after 24 hours of YC-1 treatment. In addition, we analyzed NADH autofluorescence images to quantify mitochondrial organization (β)^24^. Variations in β have been shown to be linked to alterations in bioenergetics pathways, with increases in clustering (β) attributed to hypoxia and glycolytic metabolism^21,25^. In response to YC-1 treatment, there were no changes in β in A549 or A549RR cells over the 24 hour period (p > 0.05; Fig. 2c,d), suggesting that there were no acute, time-dependent changes in mitochondrial organization following YC-1. However, these results deserve further investigation given the significant changes in the ORR and other measures of redox state.

**Figure 3:**
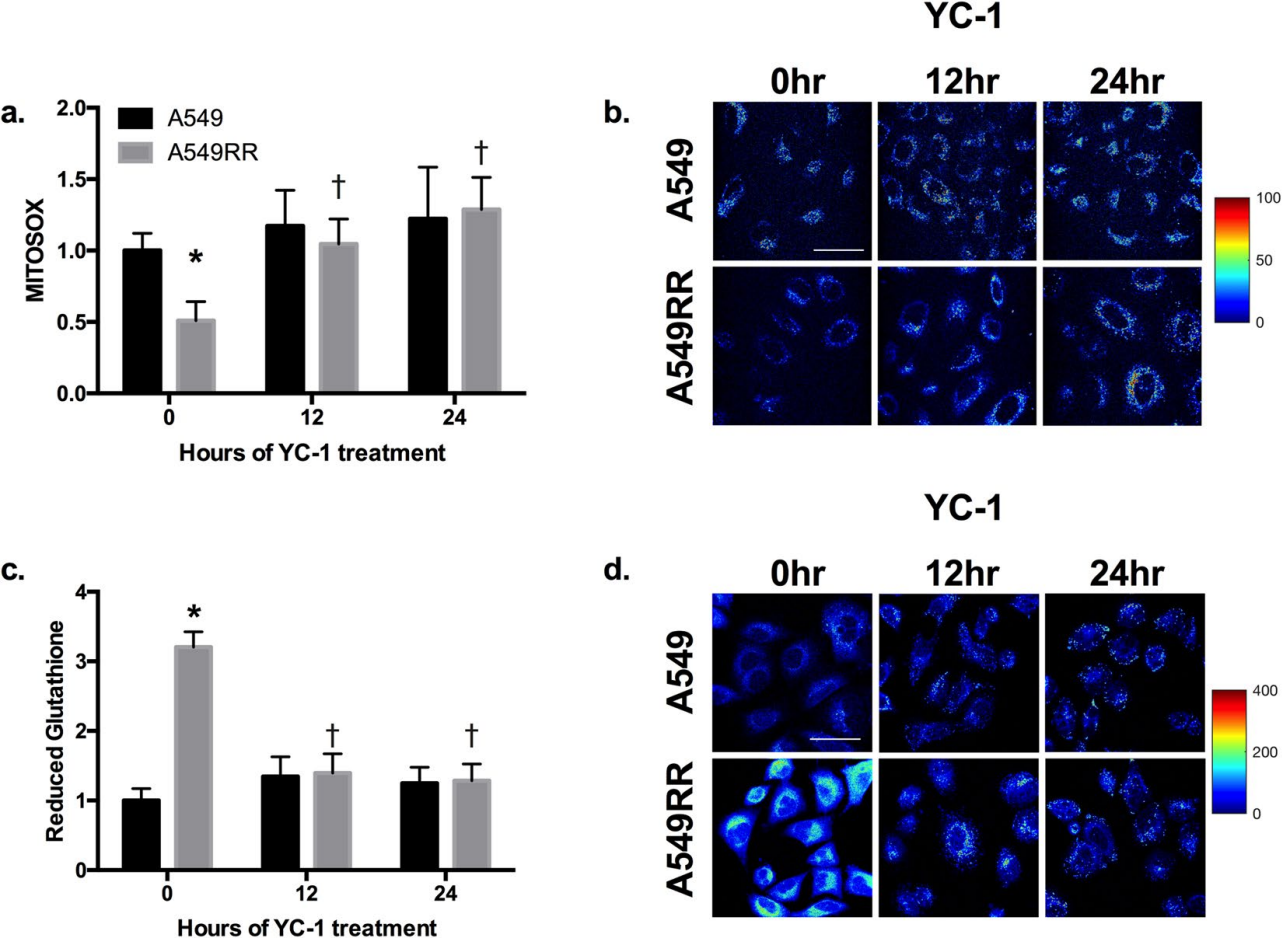
ROS and reduced glutathione are normalized in A549RR cells by HIF-1α inhibition. MitoSOX fluorescence-based mitochondrial ROS levels in A549 and A549RR cells before, 12 and 24 hours following 50 μM YC-1 treatment (**a,b**). ThiolTracker Violet was used to evaluate levels of reduced glutathione as a marker of ROS scavenging activity (**c,d**). *p < 0.05 compared to A549 0 hr control, ^†^p < 0.05 compared to same cell type at 0 hours. Scale bar in images represents 50 μm.

ROS and ROS scavenging are important factors in the cell response to radiation and stabilization of HIF-1α^26^. Therefore, we assessed mitochondrial production of ROS and levels of reduced glutathione as a measure of ROS handling. Prior to YC-1 treatment, A549RR cells had ~50% lower MitoSOX fluorescence compared to A549 (Fig. 3a; p < 0.001), with a concomitant increase in reduced glutathione (Fig. 3c; p < 0.001). Representative images of mitochondrial ROS and reduced glutathione in both cell lines at different time points are shown in Fig. 3b,d. After 12 and 24 hours of YC-1 treatment, MitoSOX levels were ~100% greater in A549RR cells compared to the A549RR-0 hour (p < 0.001) and were not significantly different from A549 parental cells (Fig. 3a,b; p > 0.05). The reciprocal pattern was seen for levels of reduced glutathione at 12 and 24 hours following YC-1 incubation with the A549RR cells normalizing to A549 (Fig. 3c,d; p > 0.05).

Previous research has identified how enhanced glycolytic metabolism can promote radiation resistance in human head and neck squamous cell carcinomas^27^. Mitigating the inherently elevated ROS production of tumor cells has also been shown to promote resistance to radiotherapy^28^. Our data build upon this evidence by demonstrating that A549RR cells have elevated glycolytic characteristics compared to A549 (increased HIF-1α, increased glucose uptake, decreased ROS production and increased ROS scavenging prior to YC-1 treatment; Figs 1–3). Furthermore, these data demonstrate that 24 hours of HIF-1α inhibition alters this metabolic profile, and forces A549RR cells towards a similar profile as the radiosensitive parental cells. To determine the effect of HIF-1α inhibition on radiation response, we elucidated the metabolic changes observed following radiation therapy alone (RT), and combined treatment with inhibition of HIF-1α followed immediately by a 2 Gy dose of radiation (Combination therapy, CT).

### CT increases glucose uptake and mitochondrial clustering

**Figure 4:**
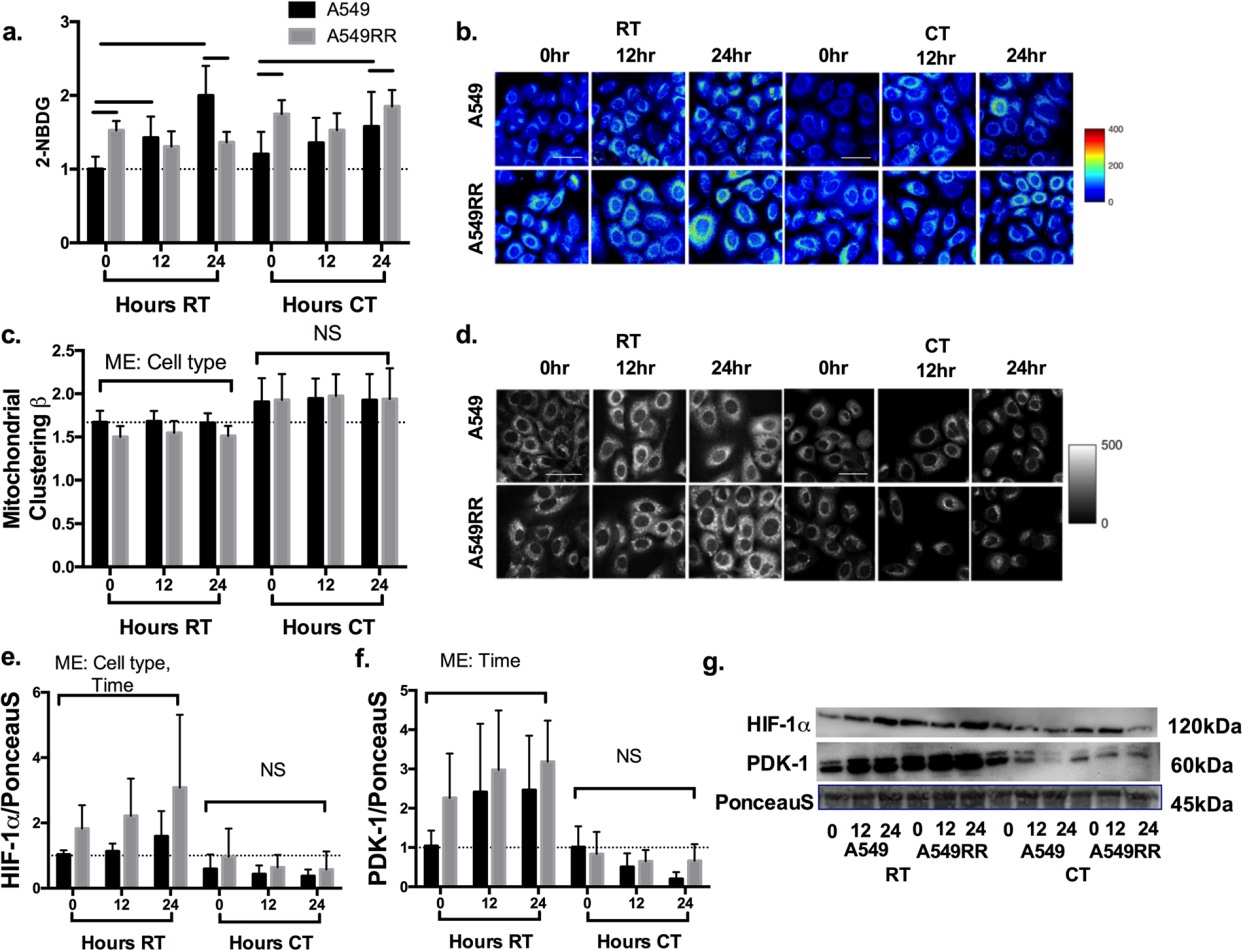
Glucose uptake and mitochondrial clustering following RT and CT. Glucose uptake (**a,b**) and mitochondrial clustering (**c,d**) was evaluated in A549 and A549RR cells before, 12 and 24 hours following radiation alone (RT) or combined radiation and YC-1 treatment. Immunoblotting was performed to verify HIF-1α and PDK-1 inhibition (**e–g**). Sample immunoblots were cropped horizontally at indicated molecular weight. Uncropped exposures can be seen in supplemental Fig. 1. Significant differences (p < 0.05) are indicated by a bar starting and ending over groups that are different. Dashed line extends across at the mean of the A549 control prior to RT and without YC-1. RT – Radiation Treatment; CT – Combination treatment; ME – Main effect only with no significant interactions present; NS – No significant differences. Scale bar in images represents 50 μm.

By analyzing the metabolic changes across 24 hours of RT side-by-side with those seen by 24 hours of CT in A549 and A549RR cells, we were able to assess time-dependent trends seen in the radiation response concurrent with radiosensitizing YC-1 treatment. Specifically, RT increased 2-NBDG in the A549 cells by ~100% over time (p < 0.001) with no significant changes in the A549RR cells over the 24 hour period (p > 0.05). Even though initial glucose uptake was greater in the A549RR cells, uptake in the A549 cells was significantly greater at the 24 hour time point (Fig. 4a,b; p < 0.001). While glucose uptake increased over time in A549 cells receiving CT (p < 0.01), uptake at 24 hours was still lower compared with the A549RR cells (Fig. 4a,b; p < 0.05). When mitochondrial organization was analyzed, clustering (β) was significantly higher in the A549 cells compared with the A549RR cells within RT groups (main effect of cell type, p < 0.001), with no significant differences seen across time (p > 0.05). Though no direct statistical comparison was made, CT led to a mean increase in clustering (β) by ~15% in the A549 cells and ~25% in the A549RR cells when compared to RT alone (Fig. 4c,d). As discussed in Fig. 2, the relationship between β and glucose catabolism within the context of radiation and chemotherapy deserves further investigation because the typical assumption of increased clustering (β) with increased glycolysis^25^ does not appear to hold up in this study. It is also possible that the changes in (β) observed within the RT and CT groups are associated with early mitochondrial changes preceding apoptosis^29^. We did not perform a direct statistical comparison between RT and CT groups because the vehicle control for YC-1 (DMSO) was not used in the RT-only experiments to create a true control. Therefore, we believe that a direct comparison might not be valid here. A549RR cells showed at least 1.8-fold greater HIF-1α content compared to A549 cells (Fig. 4e,g; p < 0.01) both prior to radiation as well as 12 and 24 hours following radiation. CT groups appeared to have a lower mean HIF-1α in both cell types and at each time point compared to RT, although this was not tested statistically for the same reasons indicated above. There were no significant differences in HIF-1α content observed between cell types or across time in CT experiments (Fig. 4e,g; p > 0.05). RT caused a > 2-fold increase in PDK-1 in the A549 cells after 24 hours relative to the 0 hour time point. PDK-1 expression was elevated in the A549RR cells compared with the A549 cells before RT (0 hours), and did not change noticeably over the 24-hour period post-RT. While statistical analysis identified a main effect of time (p < 0.05) in the RT group, there were no significant differences between cell types at any of the time points. There were no significant main effects of cell type or time on PDK-1 protein content in the CT group (Fig. 4f,g).

### ROS and ROS scavenging are normalized with CT

**Figure 5:**
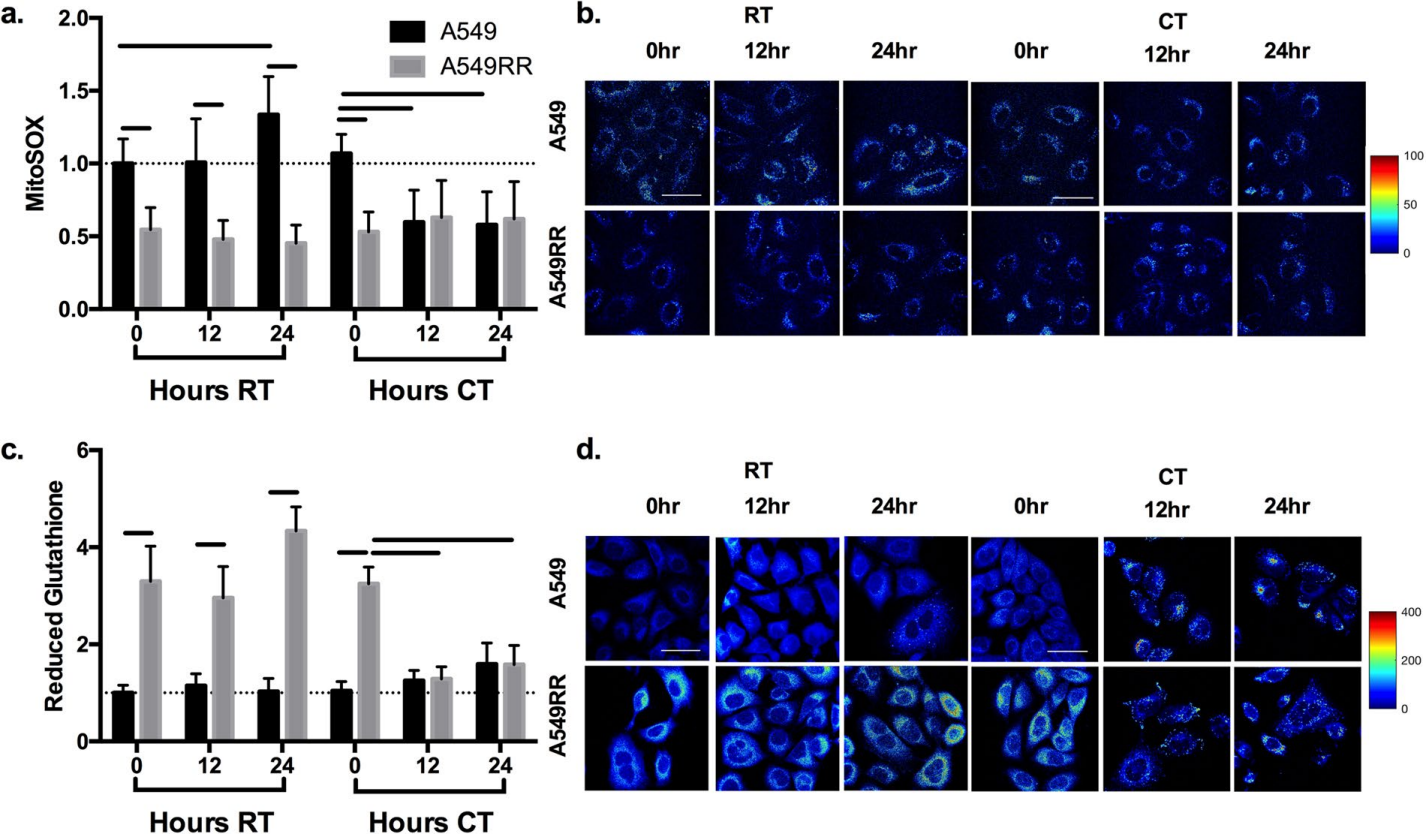
ROS and ROS scavenging following RT and CT. Mitochondrial ROS levels (MitoSOX fluorescence) in A549 and A549RR cells before, 12 and 24 hrs following radiation alone or combined radiation and YC-1 treatment (**a,b**). ThiolTracker Violet was used to evaluate levels of reduced glutathione as a marker of ROS scavenging activity (**c,d**). Significant differences (p < 0.05) between groups are indicated by a bar starting and ending over groups that are different. Dashed line extends across at the mean of the A549 control prior to RT and without YC-1. RT – Radiation Treament; CT – Combination Treatment. Scale bar in images represents 50 μm.

The decrease in reduced glutathione and an associated increased in ROS after YC-1 treatment (Fig. 3) led us to examine how radiation, alone or in combination with YC-1, affected ROS and reduced glutathione levels (Fig. 5). MitoSOX fluorescence was elevated in A549 compared to A549RR by 2-fold (p < 0.001) at each time point following RT. In addition, A549 cells demonstrated greater MitoSOX fluorescence at 24 hours following RT compared with the 0-hour time point (p < 0.01) while levels in the A549RR cells remained unchanged (Fig. 5a,b; p > 0.05). Although MitoSOX levels in the CT group were significantly higher in A549 cells compared with A549RR cells at the 0-hour time point (p < 0.001), the fluorescence levels were similar in both cell types at 12 and 24 hours following CT (Fig. 5a,b; p > 0.05). The level of reduced glutathione was 3-4.5-fold greater in A549RR throughout 24 hours of RT (p < 0.001) and at 0 hours of CT (p < 0.001); however, by 12 hours of CT, reduced glutathione levels in A549RR cells had declined to the same level as A549 (Fig. 5c,d; p > 0.05). Additionally, reduced glutathione in the A549RR cells at 12 and 24 hours following CT were significantly lower (Fig. 5c,d; p < 0.05) compared with the pre-CT time point (0 hour). While HIF-1α stabilization can be a by-product of ROS^26^, complex interactions exist between the two, including a potential feedback loop mediated by REDD1^30^. In fact, Lu *et al*.^31^ demonstrated post-chemotherapy induction of glutamate-cysteine ligase – the enzyme responsible for the γ-glutamylcysteine precursor to glutathione–in a HIF-1α dependent manner, suggesting that YC-1 may reduce the pool of glutathione available for ROS mitigation.

### ORR is sensitive to radiosensitization of resistant cells following CT

**Figure 6:**
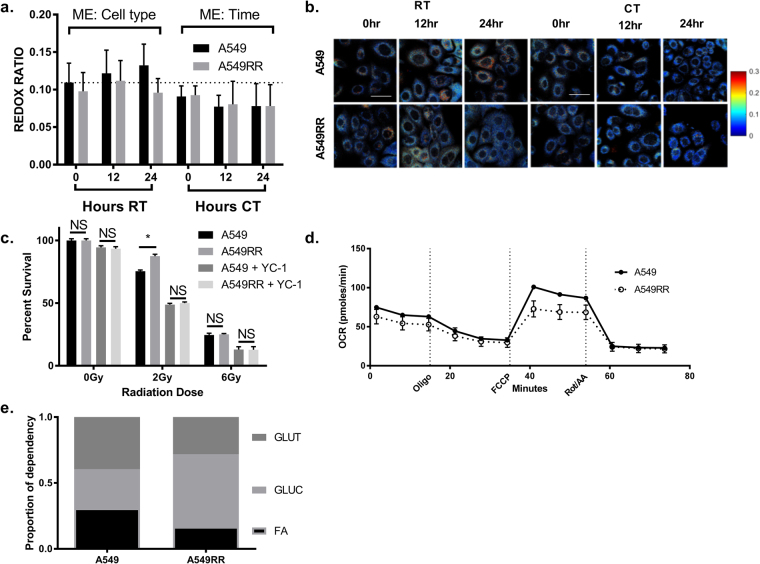
Optical redox ratio following RT and CT. Measurement of ORR in A549 and A549RR cells before, 12 and 24 hours of RT or CT (**a**,**b**). Clonogenic survival was used to determine the radiosensitizing effect of YC-1 on A549RR cells (**c**). Bioenergetic flux analysis was used to assess OCR (**d**) and fuel utilization (**e**) in A549 and A549RR cells to determine metabolic contributors to ORR. Dashed line extends across at the mean of the A549 control prior to RT and without YC-1. ME – Main effect only with no significant interactions present; RT – Radiation Treatment; CT – Combination Treatment; NS – No significant differences; GLUT – Glutamine; GLUC – Glucose; FA – Fatty Acids; Scale bar in images represents 50 μm.

The primary purpose of this investigation was not only to better understand metabolic characteristics contributing to the radiosensitizing effects of YC-1 in radiation-resistant cancer cells but to determine if the ORR can distinguish between these changes and those caused by radiation alone. While we did observe a time-dependent increase in ORR in A549 over 24 hours of RT, this was not significant (Fig. 6a,b; p > 0.05). Rather, across the 24 hours, there was a main effect of cell type on ORR (p < 0.01). Specifically, the ORR of the A549RR cells was lower than that of the A549 cells. The ORR in living cells is typically lowered during hypoxia, increased glycolytic demand, or macromolecular synthesis^14^. Taken together with the corresponding 2-NBDG data in Fig. 4a,these results suggest that factors other than changes in glycolytic demand might be responsible for the changes in ORR in the A549 and A549RR cells. With CT, ORR was unchanged between cell types (p > 0.05) but showed a significant main effect of time (Fig. 6a,b; p < 0.05). However, there were no significant differences between cell types at any of the time points. Cell survival following RT (2 Gy) was significantly greater in the A549RR cells compared with the A549 cells (Fig. 6c; p < 0.05) while CT caused a decrease in cell survival of both cell types relative to RT; in addition, there was no significant difference in cell survival between the two cell types following CT. The decrease in cell survival of the A549RR cells to the same level as the A549 cells following CT is consistent with previous work that found radiosensitization of radiation-resistant human melanoma cells following HIF-1 inhibition^32^. The decrease in cell survival following CT suggests that the decrease in ORR over time within the CT group could be due to increased apoptosis. Recent work by Heaster *et al*.^33^ showed that apoptotic cells demonstrate a greater NAD(P)H to FAD ratio.

Furdui and colleagues have shown that radiation-resistant SCC-61 head and neck squamous cell carcinoma cells maintain elevated glucose uptake despite RT by shunting glucose to the pentose phosphate pathway^34,35^. In the A549RR cells, such a glucose shunt would divert glucose away from energy production and produce excess NAD(P)H, and may be necessary due to the decreased mitochondrial oxidative capacity seen in A549RR (Fig. 6d). We have previously shown that changes in ORR are concordant with changes in the oxygen consumption rate (OCR). Therefore, although not shown in Fig. 6d, CT and RT treatment should lead to changes in the OCR that are consistent with the changes in ORR over time shown in Fig. 6a. We evaluated the specific contributions of glucose, glutamine, and fatty acids to basal mitochondrial respiration in each cell line. We found a lower reliance on glutamine and greater dependence on glucose in the A549RR cells compared with the A549 cells (Fig. 6e). These results are interesting when considered in the context of recent work from Sappington *et al*.^36^ who showed that a significant amount of glutathione in the A549 cells is derived from glutamine, and that glutamine-derived glutathione drives radiation sensitivity. The increased reliance on glucose compared with glutamine in radiation-resistant cells has also been observed in other models of radiation resistance^35^.

Any discussion of the redox ratio is incomplete without considering the possible contributions of NADPH, which cannot be distinguished from NADH^37^. The potential role of NADPH assumes greater significance within the context of radiation-induced oxidative stress. Reduced glutathione generated in the pentose phosphate pathway is an antioxidant that mitigates the effects of ROS by converting to its oxidized form. NADPH functions as an electron donor to regenerate and maintain the reduced glutathione pool. For both the A549 and A549RR cell lines, further studies using inhibitors of the pentose phosphate pathway could shed light on the specific contributions of NADPH to the overall NADH signal and hence the redox ratio. Such studies could also help better explain the trends in redox ratio seen here.

In summary, we present a label-free measure of the intracellular redox state and demonstrate the ability of this approach to identify radiation-resistant lung cancer cells within 24 hours of treatment. We used a combination of pharmaceutical HIF-1α inhibition and irradiation to sensitize resistant cells and determine the metabolic characteristics that contribute to the optical redox state. These results show that high-resolution imaging of the functional and structural changes in the mitochondria can potentially detect the radiosensitizing effects of combined treatment in radiation-resistant cancer within 24 hours. There is a considerable need for rapid, label-free methods to identify treatment options for patients undergoing radiation or chemotherapeutic treatment for cancer. These results substantiate previous evidence of utilizing optical metabolic characteristics to help identify radiation-resistant cancer^12^, and provide preliminary insights into the time-dependent changes in metabolism following radiation and combined chemoradiotherapy. Using a multiparametric imaging approach that combines fluorescence lifetime, redox ratio, and mitochondrial clustering^38^ could potentially elucidate changes to specific metabolic pathways following treatment that provide further insight into the development of resistance. As part of our future work, we plan to explore these metabolic endpoints in xenograft animal models and patient-derived tumor biopsies.

## Methods

### Cell culture, HIF-1α inhibition and irradiation

Human lung carcinoma A549 cells were incubated in Ham’s F-12K medium containing 10% FBS and 1% penicillin/streptomycin. Cells were irradiated to generate A549 cells resistant to radiation therapy (A549RR)^12^. To degrade HIF-1α, cells were treated with 50 μM (3-(5′-hydroxymethyl-2′-furyl)-1-benzyl indazole (YC-1; Sigma-Aldrich, St. Louis, MO) or equal volume DMSO as control for 0, 12, or 24 hours. For radiation treatment (RT), cells were untreated (0 hours) or exposed to 2 Gy dose of radiation and recovered for 12 and 24 hours. Radiation was performed using a biological radiator (X-Rad 320, Precision X-ray Inc., North Branford, CT). For combination treatment (CT), cell plates were first treated with 50 μM YC-1. After 5 minutes, cells were treated with 2 Gy radiation and imaged 12 or 24 hours later. A separate set of cell plates was treated with YC-1 and imaged 5 minutes later (0 hour; no RT). The radiation treatment lasts less than 2 minutes and does not add significantly to the overall timeline.

### Two-photon excited fluorescence of endogenous NADH and FAD

Two-photon excitation fluorescence was used to measure endogenous fluorescence of NADH and FAD^12,15^. Briefly, a MaiTai Ti:Sapphire laser source (Spectra-Physics, Santa Clara CA) was tuned to 755 or 860 nm for NADH and FAD excitation, respectively. Images were acquired using a resonant-galvo scanner and GaAsP photomultipler tubes (H7422-40, Hamamatsu) with bandpass filters at 440/60 nm (NADH) and 525/45 nm (FAD). Images were generated by calculating the pixel-wise FAD/(NADH + FAD) ratio normalized to 0.16 μM rhodamine B. Mitochondrial organization was evaluated through an established Fourier-based analysis of NADH images^24,25^. Images were pre-processed to minimize any scale-dependent cellular features besides mitochondria (such as cell border and nuclei) as previously described^24^. Note that such pre-processing was only performed for evaluating the mitochondrial organization. No pre-processing steps are performed prior to calculation of the redox ratio. 2D-PSD was computed from the Fourier-transformed image and radially sampled. An inverse power law model (Ak^−β^) was fit to the PSD curve corresponding to length <10 μm, and the power law exponent (β) used to measure mitochondrial clustering.

### Immunoblotting

Immunoblotting was performed as described previously^39,40^. HIF-1α (1:250 dilution, NB100-105, Novus Biologicals, Littleton, CO) and PDK-1 (1:500 dilution, Cell Signaling Technology, #3820) contents were assessed by enhanced chemiluminescence on Protein Simple Fluorochem (Minneapolis, MN) and analyzed using Images Studio Software (Li-cor Biosciences, Lincoln, NB). Images were normalized to the 45 kDa Actin band of Ponceau S as loading control. All groups were represented on each membrane and normalized to control. Sample chemiluminescent exposures are shown to aid in visualization but were horizontally cropped to include all lanes.

### Two-photon imaging of exogenous fluorophores

ThiolTracker Violet, MitoSOX Red, and 2-(N-(7-Nitrobenz-2-oxa-1,3-diazol-4-yl)Amino)-2-Deoxyglucose (2-NBDG) were purchased from Invitrogen (Eugene, OR). Cells were labeled according to manufacturer protocols and excited at 780, 960, and 970 nm, and collected using bandpass filters centered at 460/40, 525/45, and 600/70 nm, respectively.

### Bioenergetic Flux Analysis

Metabolic flux was measured using the Seahorse XFp Analyzer (Agilent, Santa Clara, CA) as previously described^12^, and according to manufacturer protocols. Cells were plated at a density of 1 × 10^4^/well using Seahorse assay media (Agilent, 103010-100) supplemented with 0.9% (w/v) glucose, 1 mM Sodium Pyruvate, and 2 mM L-Glutamine. The Agilent Seahorse XFp Cell Mito Stress Test Kit was used to acquire serial measurements of oxygen concentrations before, and following administration of 1 μM oligomycin, 1 μM FCCP, and 1 μM Rotenone and Antimycin cocktail. To determine mitochondrial fuel dependency for each of the cell lines, the Agilent Seahorse Mito Fuel Flex Test (Agilent, 103270-100) was used. Specifically, the test uses 1 μM UK5099, BPTES, or Etomoxir to inhibit mitochondrial substrate transport for glucose, glutamine, and fatty acids, respectively. This allowed determination of relative dependency on each individual pathway through analysis of oxygen consumption rates during concomitant inhibition of the two alternative fuel sources. The oxygen consumption from individual substrates was calculated as a ratio relative to total basal fuel metabolism (100%) and displayed as a proportion of total oxygen utilization for each substrate.

### Clonogenic Survival Assay

To determine survival following RT, 100 cells were seeded in 9 cm^2^ dishes, incubated overnight, and exposed to RT. Following exposure to 0, 2, or 6 Gy radiation, the cells were incubated for 8 days, fixed in methanol and stained using crystal violet. The number of colonies per well was determined and normalized to control.

### Statistical Analysis

Cell culture experiments were performed in triplicate on multiple independent technical replicates. A 2 × 3 repeated measures ANOVA was used to compare cell types (A549 or A549RR) and time following treatment (YC-1, RT, or CT) for 0, 12, and 24 hours. Where significant interactions were found between the two main effects (ME) of cell type and time after treatment, Tukey’s Honest Significant Difference (HSD) post-hoc analysis evaluated differences between groups, and significant differences are indicated by a horizontal bar between the two groups. To determine significance, α was set at 0.05. NS in figures represents data where no statistical main effect of cell type, time after treatment, or interaction between these two effects was determined at α = 0.05. RT only and combined treatment with YC-1 and RT (CT) were not compared statistically but graphed together for visual comparisons. For bioenergetics experiments where A549 were compared to A549RR, Student’s t-test was used. Results are presented as mean ± STD. Statistical analyses were performed using GraphPad Prism 6. The datasets generated during and/or analyzed during the current study are available from the corresponding author upon reasonable request.

## Electronic supplementary material

Supplementary Figure 1
