# Supplementary material for: A Radiosensitizing Inhibitor of HIF-1 alters the Optical Redox State of Human Lung Cancer Cells In Vitro
Source: Sci Rep. 2018 Jun 11;8:8815. doi: 10.1038/s41598-018-27262-y (PMC5995847; doi:10.1038/s41598-018-27262-y)
Supplement: Supplementary file 1 — Supplementary Figure 1 [file 41598_2018_27262_MOESM1_ESM.pdf]

Title: **A Radiosensitizing Inhibitor of HIF-1 alters the Optical Redox State of Human Lung Cancer Cells *In Vitro***

Short Title: Optical Redox Imaging during Radiosensitization

Authors: David E. Lee,[ac]* Kinan Alhallak,[a]* Samir V. Jenkins,[b] Isaac Vargas,[a] Nicholas P. Greene,[c] Kyle P. Quinn,[a] Robert J. Griffin,[b] Ruud P. M. Dings,[b] and Narasimhan Rajaram[a]

[a]Department of Biomedical Engineering, University of Arkansas, Fayetteville, AR, 72701
[b]Department of Radiation Oncology, University of Arkansas for Medical Sciences, Little Rock, AR, 72205
[c]Department of Health, Human Performance, and Recreation, University of Arkansas, Fayetteville, AR, 72701
* - Authors contributed equally to the work

Keywords: Redox ratio, YC-1, radiation therapy, Two photon excitation fluorescence, Optical metabolic imaging

Corresponding Authors: Narasimhan Rajaram (nrajaram@uark.edu) and Ruud P. M. Dings (rpmdings@uams.edu)

Department of Biomedical Engineering

700 W. Research Center Blvd.

Fayetteville, AR 72701

Phone: (479)575-7696

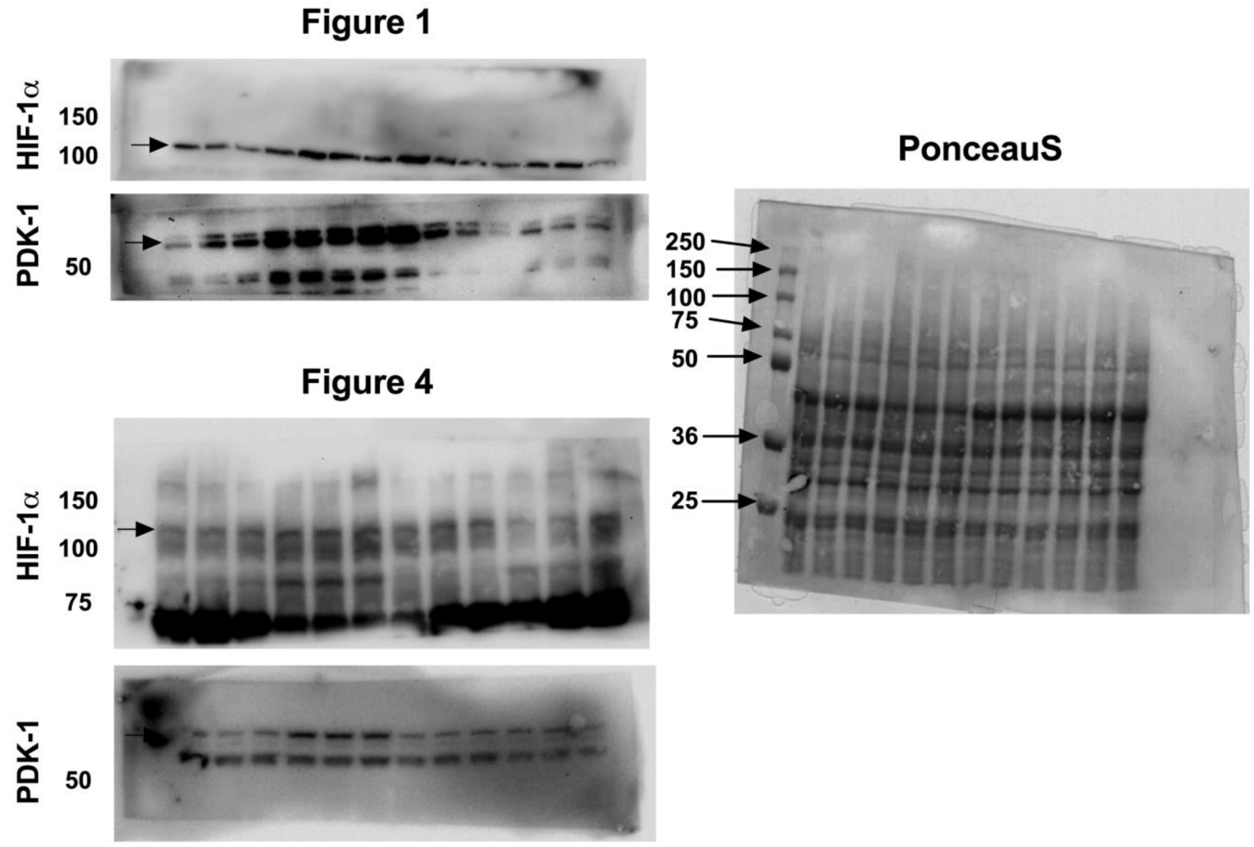

Supplemental Figure 1. Sample Immunoblot exposures. Samples immunoblot images seen in the primary figures have been horizontally cropped from original image exposures. Original HIF-1α and PDK-1 immunoblot chemiluminescent exposures are shown as represented by cropped images in Figures 1 and 4. Example white light image (converted to grayscale) of Ponceau S stain of transferred protein alongside molecular weight ladder. Membranes were physically cut horizontally at or near the 75kDa marker following Ponceau S staining to allow multiple antigen targets on a single membrane. For chemiluminescent images, molecular weight ladders were visualized under white light and superimposed to verify the correct band was analyzed. On the left images, arrows indicate band that was analyzed for final analysis. For the Ponceau S image, arrows indicate molecular weight ladder size.
